# Editorial: Remote Ischemic Conditioning (Pre, Per, and Post) as an Emerging Strategy of Neuroprotection in Ischemic Stroke

**DOI:** 10.3389/fneur.2022.932891

**Published:** 2022-06-23

**Authors:** Francisco Purroy, Simone Beretta, Timothy J. England, David Charles Hess, Fernando Pico, Ashfaq Shuaib

**Affiliations:** ^1^Clinical Neurosciences Group IRBLleida, Stroke Unit, Hospital Universitari Arnau de Vilanova de Lleida, Universitat de Lleida, Lleida, Spain; ^2^Department of Neurology, San Gerardo Hospital Monza, Monza, Italy; ^3^Stroke Trials Unit, University of Nottingham, London, United Kingdom; ^4^Medical College of Georgia, Augusta University Augusta, Augusta, GA, United States; ^5^Centre Hospitalier de Versailles Le Chesnay, Le Chesnay, France; ^6^Department of Neurology, Medical College of Georgia, University of Alberta Edmonton, Edmonton, AB, Canada

**Keywords:** remote ischemic conditioning, remote ischemia postconditioning, remote ischemia preconditioning, remote ischemia perconditioning, ischemic stroke, neuroprotection

Stroke is one of the leading causes of death and disability worldwide (1–3). Currently, the only treatments available in the acute phase demonstrating safety and effectiveness are intravenous fibrinolytic treatment and mechanical thrombectomy (4). Unfortunately, many patients cannot benefit from these treatments due to contraindications, time of evolution of the symptoms, or restricted access to mechanical therapies that are currently only offered in specialized centers (5). The efficacy of neuroprotective therapies has great potential, although translation of most neuroprotective trials from the bench to bedside has failed so far (6, 7).

Remote ischemic conditioning (RIC) represents a new paradigm in neuroprotective therapies (8–10), and it has the potential ability to protect the ischemic brain from injury until reperfusion and, later, to protect the brain from reperfusion injury (8, 11). RIC consists of short and controlled cycles of ischemia-reperfusion applied to one limb during the establishment of cerebral ischemia (perconditioning), before (preconditioning), or after (postconditioning) (11). Until now, the underlying mechanisms of RIC are not clear and there are limited data about the clinical translation of RIPerC in ischemic stroke patients (8, 11). Recent trials have only demonstrated the feasibility and safety of this intervention in acute ischemic stroke patients (AIS) (12–16).

In this special issue, we provide new insights into the mechanisms of RIC in ischemic stroke Abbasi-Habashi et al. and Pignataro we compare evidence of the effect of RIC in AIS and myocardial infarction Saccaro et al. Furthermore, we propose new indications or aims for the use of RIC in AIS as preventing further ischemic cerebrovascular events Liu et al. or stroke-associated pneumonia Zhang et al. and improving cognition Poalelungi et al.. Finally, we identify or we propose to identify new subgroups of patients who could benefit from this neuroprotective strategy such as Moyamoya disease Xu et al. and AIS who are not eligible for recanalization therapies Diamanti et al..

Although the exact mechanism by which the protective signal of RIC is transferred from the arms or limbs to the brain remains unclear, preclinical studies suggest that a combination of circulating humoral factors and neuronal signals is involved. RIC could improve the outcomes of AIS treated with reperfusion therapies by reducing reperfusion injury (17). In this issue, Abbasi-Habashi et al. review the putative role of the immune system and circulating mediators of inflammation in these protective processes and the potential role of extracellular vesicles. In this line, Pignataro discusses the role of miRNAs in the activation of endogenous tolerance mechanisms by RIC as transducers of protective messages to the brain and/or as effectors of brain protection.

Myocardial infarction (MI) and AIS have some similarities. Both conditions have an acute onset due to blood vessel occlusion and RIC has been proposed as a strategy to improve reperfusion therapies. Ischemic tolerance and RIC have been first described in MI (11). Clinical trials also started earlier in MI (11) than in AIS patients (9). Saccaro et al. explore similarities and differences of the response of RIC in both conditions. RIC reduces circulating biomarkers of myocardial necrosis, infarct size, and edema although these effects appear to have no effect in the outcomes of MI patients (18). In AIS, RIC is also effective in pre-clinical models (8) but has no significant clinical evidence in the few small studies completed to date (10). The lack of conclusive clinical evidence of RIC efficacy in MI and AIS may be due to heterogenous protocols and different RIC applications. They recommend improving the selection criteria in future RIC clinical trials. Based on pre-clinical studies that demonstrate a greater effect of RIC against reperfusion injury and on its effect in enhancing cerebral collateral circulation, they propose to focus on patients with large vessel occlusion who are candidates for mechanical thrombectomy and can most benefit from the presence of vascular collaterals Saccaro et al.. However, there are still proposals to study the effect of RIC in other subgroups of patients. Diamanti et al. designs the multicenter phase II study TRICS-9 to assess the efficacy of RIC in patients with AIS within 9 h of onset who are not candidates for recanalization therapies.

Repeated RIC post-conditioning (RIPostC) emerges as a promising strategy to improve functional recovery (13). In this special issue, different studies investigate outcomes and indications for RIPostC. Poalelungi et al. in a single center double-blind randomized controlled trial observed that RIPostC during 5 days of hospitalization twice daily might improve disability and cognition at 180 days. Interestingly, Liu et al. in a single-arm open-label phase IIa futility trial (PICNIC-One study) applied RIPostC twice a day for 90 days in 167 acute minor ischemic stroke or moderate-to-high risk transient ischemic attack patients, which seemed to reduce the risk of recurrent stroke. However, only 42% of subjects completed ≥50% of 45-min RIC sessions. The compliance of patients to RIPostC for several days or months could be important to guarantee its protective effect. Zhao et al. investigate the factors that influence compliance to long-term RIC. The number of follow-up visits and physiological discomfort associated with RIPostC treatment independently influenced patient compliance. Xu et al. in a small study analyses the effect of RIPostC for 1 year among Moyamoya disease patients. They observe improving cerebral blood flow and slowing arterial progression of the stenotic-occlusive lesions. Finally, Zhang et al. evaluated RIPostC over 6 days in the prevention for stroke-associated pneumonia (SAP) in a “proof of concept” pilot randomized controlled trial. According to these authors the possible anti-inflammatory effect of RIC could prevent SAP. Although proinflammatory cytokines levels at day 5 after admission were significantly lower in the RIPostC group than in the control group, no clinically significant effect was observed, possibly due to the small size of the trial.

RIC is a non-invasive, simple, safe, and cheap neuroprotective strategy with multiple mechanisms of action. Its clinical efficacy in acute ischemic stroke patients remains to be proven. RIPostC seems to be the most effective modality of RIC. Therefore, future trials could focus on patients with large vessel occlusion who are candidates for mechanical thrombectomy. Chronic, daily RIPostC could be an option to reduce stroke recurrence in high-risk patients, to improve disability and cognition after AIS, and to improve cerebral perfusion in Moyamoya disease.

## Author Contributions

FP wrote the first draft. All authors could review it and did their own contributions.

## Funding

This study was funded by Carlos III Health Institute and co-funded by European Union (ERDF A way to make Europe) Project (PI17-01725) and the RICORS Research Network to FP, NIH Funding (R01 NS099455, 1UO1NS113356, and R01 NS112511) to DH, Italian Ministry of Health - PRIN 2017CY3J3W to SB. French National Minsitry of Health Grant 2014 AOR13032 to FP, TE is the Chief Investigator for the Remote ischaemic conditioning after stroke trial (RECAST), RECAST-2, and RECAST-3 funded through the NIHR Efficacy and Mechanism Evaluation (EME) Programme, Award ID NIHR128240.

## Conflict of Interest

The authors declare that the research was conducted in the absence of any commercial or financial relationships that could be construed as a potential conflict of interest.

## Publisher's Note

All claims expressed in this article are solely those of the authors and do not necessarily represent those of their affiliated organizations, or those of the publisher, the editors and the reviewers. Any product that may be evaluated in this article, or claim that may be made by its manufacturer, is not guaranteed or endorsed by the publisher.
